# Friction on water sliders

**DOI:** 10.1038/s41598-019-40797-y

**Published:** 2019-03-11

**Authors:** Giuseppe Pucci, Ian Ho, Daniel M. Harris

**Affiliations:** Brown University, School of Engineering, 184 Hope St., Providence, (RI) United States

## Abstract

A body in motion tends to stay in motion but is often slowed by friction. Here we investigate the friction experienced by centimeter-sized bodies sliding on water. We show that their motion is dominated by skin friction due to the boundary layer that forms in the fluid beneath the body. We develop a simple model that considers the boundary layer as quasi-steady, and is able to capture the experimental behaviour for a range of body sizes, masses, shapes and fluid viscosities. Furthermore, we demonstrate that friction can be reduced by modification of the body’s shape or bottom topography. Our results are significant for understanding natural and artificial bodies moving at the air-water interface, and can inform the design of aerial-aquatic microrobots for environmental exploration and monitoring.

## Introduction

Friction is the force that opposes the relative motion of solid bodies and fluids and can present itself in both dry and fluid forms. For the case of fluid friction, a body moving with speed *U* with respect to the surrounding fluid generally may experience three primary types of drag: Stokes drag, form drag and skin friction. The relative importance of Stokes drag and form drag depends on the Reynolds number *Re* = *UL*/*ν*, where *L* is the body size and *ν* is the fluid kinematic viscosity, and which represents the ratio between the fluid inertia and viscous stresses. Stokes drag tends to dominate for small *Re* and is proportional to *U*, while form drag is significant for larger *Re* and is proportional to *U*^2^. Another component of hydrodynamic resistance is the skin friction due to the viscous boundary layer that forms in the vicinity of the body surface. In the case of unseparated laminar flow on a flat plate ($Re\lesssim {10}^{6}$), skin friction is proportional to *U*^3/2^ ^1^. Quantifying these types of friction is of critical importance for the design of boats, robots, and projectiles as well as for understanding the motion of living organisms in water and air. In this report, we demonstrate that the dominant friction for centimetric, hydrophobic bodies sliding on the surface of water is skin friction and provide a simple model which rationalizes our observations. Our findings are particularly relevant for the study of natural^2^ and artificial bodies self-propelling at the water-air interface. Natural systems include water striders, which move on the water surface by generating vortices^3^ with their super-hydrophobic legs^4^, fisher spiders^5^, meniscus-climbing insects^6^, and insects that self-propel by generating surface-tension gradients^7^. Artificial systems include magnetic^8^ and magneto-capillary^9^ swimmers, droplets^10,11^ and boats^12,13^ propelled by surface-tension gradients, droplets propelled by surface waves^14–16^, which can find application in micro-manipulation and micro-transport and also can constitute the building blocks for artificial active-matter systems at the fluid interface. Moreover, our findings are relevant for the design of robots^3,17–22^ and microrobots^23,24^ at the water-air interface for environmental monitoring and exploration.

## Results

**Figure 1:**
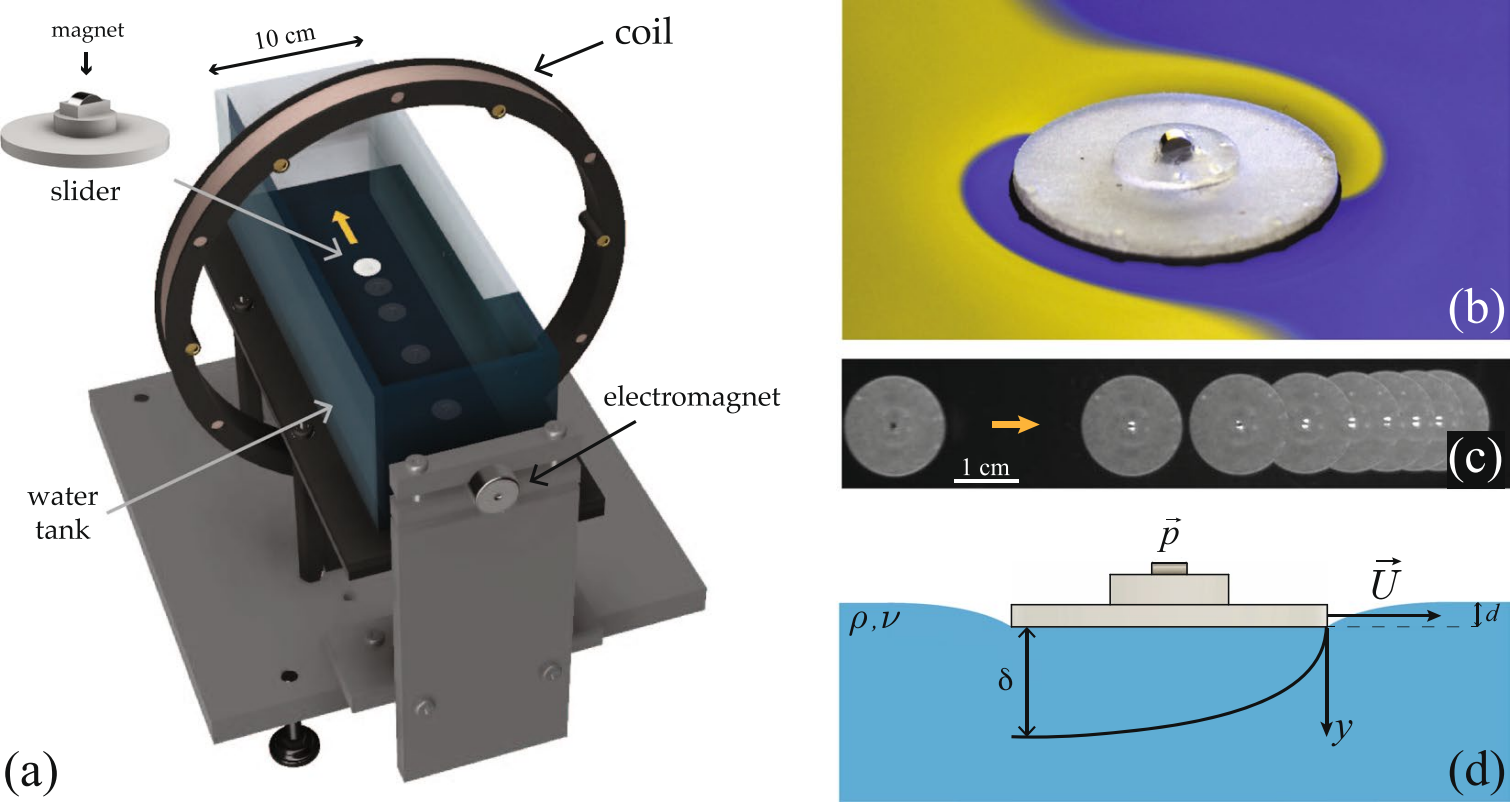
(**a**) CAD rendering of the experimental setup. A coil and an electromagnet allow for control of the slider on the water surface and continuous run of the experiment (Supplementary Movie). (**b**) Photograph of a slider with radius *R* = 8.00 ± 0.05 mm floating on water. Colours are obtained from the distorted reflection of a yellow and blue background on the water surface. (**c**) Image sequence of a decelerating slider with radius *R* = 8.00 ± 0.05 mm. Frames are taken 1 second apart. (**d**) Side view schematic of the slider motion and the boundary layer generated in the underlying fluid (not to scale).

In our experiments sliding bodies, henceforth “sliders” (Fig. 1(b)) were supported at the water-air interface by virtue of the equilibrium between their weight, surface tension, and hydrostatic forces resulting in a maximum interface deformation *d*. The maximum deformation of the free surface over all trials is estimated to be approximately 12% of the slider’s diameter. A magnet was embedded in the sliders, which were accelerated along a straight line on the surface of a water bath through an inhomogeneous magnetic field generated by a coil (Fig. 1(a)). The coil was then turned off and the slider decelerated due to fluid friction (Fig. 1(c)).

The deceleration phase was recorded and the slider’s speed as a function of time was measured. Our setup allowed for continuous and repeatable runs of the experiment, which we show in the Supplementary Movie and describe  in the Supplementary Materials. Extended details of the experimental setup can be found in the Methods section. The slider speed at the beginning of the deceleration phase was always in the range *U*_0_ = 6.1–16.0 cm/s with uncertainty 0.1 cm/s, below the phase speed of capillary waves ${U}_{c}\simeq 23$ cm/s, at which the slider with constant speed would generate a capillary wake^25^. Visible capillary waves were generated during the initial acceleration period^26,27^, however no additional waves were observed during the deceleration phase over which our measurements take place.

**Figure 2:**
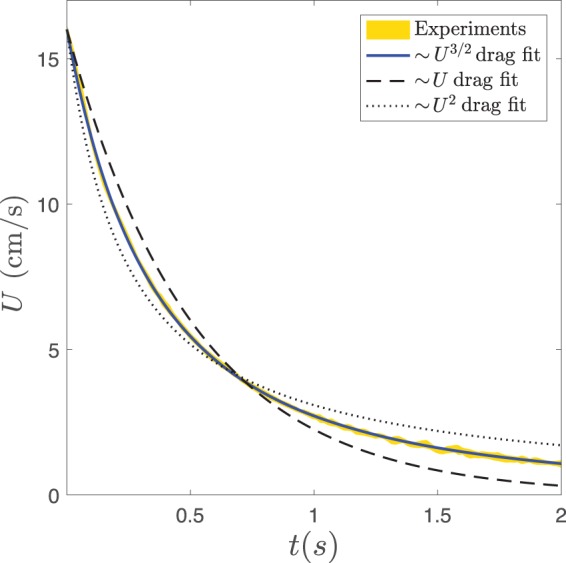
(**a**) Attempt to fit a sample data set with *U*(*t*) as a solution to *mdU*/*dt* = −*F*_*D*_ when three different drag forces are considered. *U*(*t*) = *U*_0_ exp {−*ct*} when *F*_*D*_ ∝ *U*, *U*(*t*) = 1/(1/*U*_0_ + *ct*) when *F*_*D*_ ∝ *U*^2^ and $U(t)=\mathrm{1/}{(\mathrm{1/}\sqrt{{U}_{0}}+ct)}^{2}$ when *F*_*D*_ ∝ *U*^3/2^, where *c* is the fitting parameter and *U*_0_ the measured initial speed. The shaded area represents the mean speed over at least 39 runs to within two standard deviations. The slider has radius *R* = 4.00 ± 0.05 mm and mass *m* = 61 ± 2 mg, and the fluid kinematic viscosity is *ν* = 0.0100 ± 0.0002 cm^2^/s.

In the deceleration phase, the slider obeys the simple ordinary differential equation (o.d.e.) *m* *dU*/*dt* = −*F*_*D*_, where *U* is the slider speed and *F*_*D*_ is a drag force. We proceeded by testing the solutions *U*(*t*) of the o.d.e. as obtained when three common fluid drag scalings are considered: Stokes drag *F*_*D*_ ∝ *U*, form drag *F*_*D*_ ∝ *U*^2^ and skin friction *F*_*D*_ ∝ *U*^3/2^. The results are presented in Fig. 2 and show evidently that the skin friction-type scaling completely captures the observed deceleration behavior. This is consistent with the order-of-magnitude estimate presented in the discussion section.

Skin friction is due to the viscous stress developed in the liquid boundary layer underneath the slider (Fig. 1(d)). We now show that in our experiments the boundary layer can be considered as quasi-steady. In our experiments, 40 < *Re*_*R*_ < 3 · 10^3^, where *Re*_*R*_ = *U*_0_*R*/*ν* is the Reynolds number, which suggests laminar flow. The typical timescale *t*_*BL*_ over which the boundary layer develops can be derived by balancing fluid inertia and viscous stress

1 $$\rho \frac{\partial U}{\partial t}\sim \mu \frac{{\partial }^{2}U}{\partial {y}^{2}},$$

where *μ* = *ρν* is the liquid dynamic viscosity. A scaling analysis of Eq. 1 gives *ρU*_0_/*t*_*BL*_ ~ *μU*_0_/*δ*^2^, where $\delta \sim R/\sqrt{R{e}_{R}}$ is the boundary layer thickness from laminar boundary layer theory^1^. This yields *t*_*BL*_ ~ *R*/*U*_0_^28^, which can be interpreted as an advection timescale. In the acceleration phase, the slider accelerates over a time *t*_*acc*_ ~ 1 s, larger than *t*_*BL*_ ~ 0.1 s. We thus conclude that the boundary layer is already fully developed at the start of the deceleration phase. The characteristic timescale of slider deceleration can be derived from the balance between the slider’s inertia and viscous stress

2 $$m\frac{dU}{dt}\sim {\tau }_{w}\,A$$

where *A* is the contact area between the slider and the water surface and ${\tau }_{w}\sim \mu \frac{\partial U}{\partial y}$ is the shear stress at the fluid interface. A scaling analysis of Eq. 2 gives *mU*_0_/*t*_*d*_ ~ *ρνU*_0_*R*^2^/*δ*, where we considered the initial slider’s speed *U*_0_ as typical speed for both slider and fluid, *A* ~ *R*^2^ for a circular slider and *t*_*d*_ is a characteristic timescale. This balance ultimately allows us to define a characteristic timescale of deceleration

3 $${t}_{d}\equiv \frac{m}{\rho \sqrt{\nu {U}_{0}{R}^{3}}}.$$

**Figure 3:**
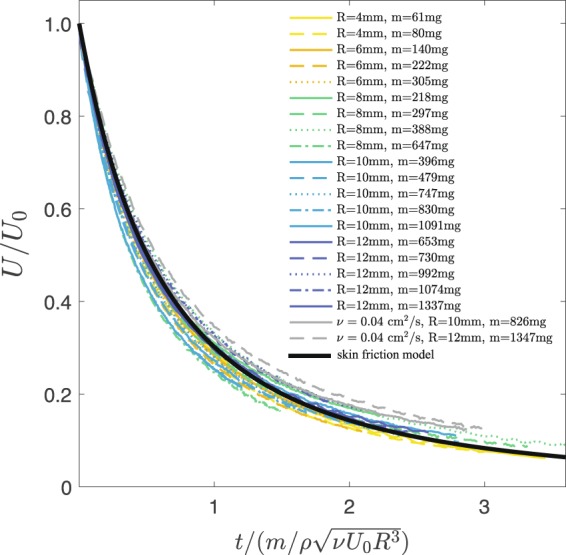
Collapse of data spanning different slider radii *R*, masses *m*, and fluid viscosities *ν*. Lines are averages over at least 39 runs and are compared to the theoretical expression *U*(*t*)/*U*_0_ = 1/(1 + 1.64*t*/2*t*_*d*_)^2^, where ${t}_{d}=m/\rho \sqrt{\nu {U}_{0}{R}^{3}}$ (black line). Unless otherwise specified, the fluid kinematic viscosity is *ν* = 0.01 cm^2^/s. Uncertainty is 0.0002 cm^2^/s on the kinematic viscosity due to variations in fluid temperature, 0.05 mm on the radii and 2 mg on the mass.

In all of our experiments ${t}_{d}\gg {t}_{BL}$, which allows us to treat the boundary layer as quasi-steady. In other words, in the deceleration phase the boundary layer continuously adjusted to the slider speed over a timescale that is much smaller than the slider deceleration timescale. We validated this hypothesis by varying a number of system parameters, including slider radius *R* = 4–12 mm, mass *m* = 54–1337 mg and liquid viscosity *ν* = 0.01–0.04 cm^2^/s. Collapse of the data using the characteristic time *t*_*d*_ and speed *U*_0_ is presented in Fig. 3 and it is effective for all slider radii, masses and fluid viscosities considered. The o.d.e. describing the system is thus

4 $$m\frac{dU}{dt}=-\,\beta {U}^{\mathrm{3/2}},$$

where *β* is the friction coefficient. We calculate *β* by using the classical Blasius solution derived for a steady boundary layer in a laminar flow beneath a semi-infinite and one-dimensional flat plate^1^. Under these assumptions, the drag force per unit width on one side of a plate of length *L* is ${f}_{D}=0.664\,\rho {U}^{2}L/\sqrt{R{e}_{L}}$. We integrate this force over the slider surface in contact with the bath and obtain $\beta =\alpha \rho \,\sqrt{\nu }{R}^{\mathrm{3/2}}$, thus the friction force on the slider can be written as

5 $${F}_{D}=\alpha \rho \sqrt{\nu }\,{R}^{\mathrm{3/2}}{U}^{\mathrm{3/2}},$$

with *α* = 1.64. In our experiments, the friction force is in the range *F*_*D*_ = 0.025–8.2 dynes (0.25–82 *μ*N). The solution to Eq. 4 can thus be obtained by integration and written as

6 $$\frac{U(t)}{{U}_{0}}=\frac{1}{{\mathrm{(1}+\alpha t\mathrm{/2}{t}_{d})}^{2}}.$$

This solution is plotted on Fig. 3. The agreement with experimental data is satisfactory despite the assumptions in the theory and agrees up to a factor of order 1 on the friction coefficient *β*. In fact, by fitting each data set with Eq. 6 and with *α* as a free parameter, we obtain *α* = 1.3–2.1, close to the theoretical value *α* = 1.64. Values of *α* for specific circular sliders are reported in the Supplementary Table.

**Figure 4:**
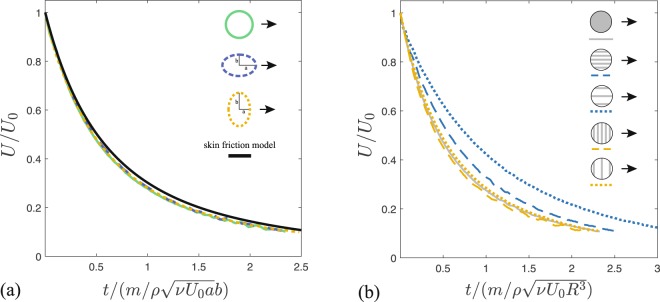
(**a**) Collapse of data from a circular slider of radius *R* = 4.00 ± 0.05 mm and mass *m* = 61 ± 2 mg, and two elliptical sliders with the same surface area. One elliptical slider slid with its major semi-axis *a* = 5.00 ± 0.05 mm parallel to the direction of motion and has mass *m* = 57 ± 2 mg, while the other slid with it minor semi-axis *a* = 3.20 ± 0.05 mm parallel to the direction of motion and has mass *m* = 54 ± 2 mg. *b* denotes the semi-axis perpendicular to the direction of motion. (**b**) Comparison of data from circular sliders with and without a grooved bottom. Sliders with ridges parallel to the direction of motion experience less friction than a slider without ridges or with ridges perpendicular to the direction of motion. Friction decreases further by increasing the spacing between the ridges. Sliders were all of radius *R* = 4.00 ± 0.05 mm and the masses were in the range *m* = 76–86 mg with uncertainty 2 mg.

We then explored the possibility of modifying the friction properties by changing the slider shape and its orientation with respect to its direction of motion. Elliptical sliders were slid with their major axis parallel or perpendicular to the direction of motion. The slider contact surface was chosen to equal the contact surface of a circular slider of radius *R* = 4.00 ± 0.05 mm. Comparison between elliptical, circular sliders and theoretical prediction is presented on Fig. 4(a). For elliptical sliders, the deceleration time can be computed again from the balance between the slider’s inertia and viscous stress (now with *A* ~ *ab*) which yields ${t}_{d}=m/\rho \sqrt{\nu {U}_{0}a}b$, where *a* is the semi-axis parallel to the sliding direction and *b* is the semi-axis perpendicular to the sliding direction. Alternatively, one can obtain the theoretical force expression by integrating the drag force per unit width over the elliptical domain. The collapse of the experimental data is excellent and the agreement with the theoretical prediction is satisfactory (Fig. 4(a)). Fitting of the parameter yields *α* = 1.7–1.8 in all experimental configurations (ellipses and circular), again close to the expected theoretical value *α* = 1.64. We note that the timescale of deceleration *t*_*d*_ of the elliptical slider sliding with its major axis parallel to the direction of motion is larger, showing that the friction depends explicitly on the shape and the orientation of the slider. The elliptical slider with major axis along the direction of motion shows the longest deceleration time, and thus a decreased friction.

We then investigated the possibility of modifying skin friction by changing the topography of the slider bottom surface in contact with the fluid interface. Recent experiments on centimetric boats with super-hydrophobic, grilled bottoms, have shown that the drag on these boats can be reduced with respect to boats with flat bottoms^29^. We further explored this concept by performing experiments on circular sliders with grooved bottoms, in which the ridges were either parallel or perpendicular to the sliding direction (Supplementary Figure). The ridges lifted the slider above the interface and thus air could flow within the macroscopic grooves. Results are presented in Fig. 4(b). We found that ridges parallel to the direction of motion significantly reduce the skin friction, with the friction being lower as the spacing between ridges is increased. Surprisingly, sliders with ridges perpendicular to the direction of motion behave very similarly to sliders with flat bottom. This clearly demonstrates that the contact area is not the only parameter that matters, but the orientation of the body also has a dramatic effect on the friction of the slider.

## Discussion

Our experiments demonstrate that centimetric bodies sliding at an air-water interface are decelerated by skin friction, which is dominant with respect to other forms of hydrodynamic drag. We can also understand this result by estimating the relative importance of Stokes drag and form drag with respect to skin friction. The Reynolds number in the liquid was always 40 < *Re*_*R*_ < 3 · 10^3^, $R{e}_{R}\gg 1$. Moreover, the depth of the bath *h* = 3.7 cm was chosen much larger than the maximum expected thickness ${\delta }_{max}\simeq 5\sqrt{2{\nu }_{m}R/{U}_{min}}\simeq 1.6$ cm of the boundary layer that would develop underneath the slider^1^, where *ν*_*m*_ = 0.04 cm^2^/s is the maximum kinematic viscosity explored and *U*_*min*_ = 1.0 cm/s is the minimum slider speed considered. We thus operated in the deep water regime avoiding the possibility of a Stokes (lubrication) regime in shallow water. The friction force is thus contributed by air drag *F*_*air*_ and skin friction *F*_*skin*_. Skin friction in the regime of laminar flow scales as ${F}_{skin}\sim \rho \sqrt{\nu }\,{R}^{\mathrm{3/2}}{U}^{\mathrm{3/2}}$ ^1^. Air drag scales as ${F}_{air}\sim {\rho }_{a}{u}^{2}R{h}_{s}$, where *ρ*_*a*_ = 1.2 · 10^−3^ g/cm^3^ is the density of air and *h*_*s*_ ~ 0.1 cm is the typical slider thickness. In our experiments, *F*_*air*_/*F*_*skin*_ ~ 10^−3^, thus air drag is negligible with respect to skin friction.

Finally, we discuss the condition under which we expect our quasi-steady theory to remain applicable. In our experiments, the typical deceleration time ${t}_{d}=m/\rho \sqrt{U\nu {R}^{3}}$ is much larger than the time required for the boundary layer to set up *t*_*BL*_ = *R*/*U*, where *U* is the instantaneous body speed. From this, we define a “sliding number” as the ratio *t*_*BL*_/*t*_*d*_, which yields

7 $$Sl=\frac{\rho }{m}\sqrt{\frac{{R}^{5}\nu }{U}}.$$

We remark that the sliding number may also be interpreted as the ratio between the fluid inertia *I*_*BL*_ ~ *ρR*^2^*δU* and the body inertia *I*_*b*_ ~ *mU*. A small sliding number is the condition for the free-sliding behaviour to be adequately described by our quasi-steady boundary layer model. In all of our experiments, *S*_*l*_ < 0.3 and the experimental behaviour is well captured by a simple model that considers the laminar boundary layer underneath the slider as quasi-steady. The model appears to apply to different slider shapes, and we have shown that shapes elongated in the direction of motion yield lower overall friction. The free surface deformation due to the slider’s weight appears not to play a significant role in the process.

Skin friction can be further controlled by modifying the topography of the slider bottom, specifically if the bottom has grooved recessions parallel to the direction of motion. As the contact surface in topographic sliders is lower than in the full contact sliders, one might expect friction to be reduced for all topographic sliders. However, we find that friction reduction depends on the ridge orientation. This presumably results from the shear stress in a developing boundary layer being largest at the leading edge of any solid surface. Therefore, sliders with ridges perpendicular to the direction of motion have a larger projected ridge width. Furthermore, the boundary layer must restart in some manner at each successive ridge, again leading to additional drag as compared to the sliders with grooves parallel to the direction of motion.

Our work opens the door to further experimental and theoretical investigation of the friction experienced by small bodies moving on the surface of water as a function of their shape and bottom topography, with motivations of either drag reduction or propulsive efficiency through enhanced traction. Moreover, the experimental setup we have developed can be adapted for studying the drag on partially submerged centimetric bodies or to investigate the effect of an unsteady boundary layer^28,30^, which is expected to become significant at sliding number $Sl\gtrsim 1$.

## Methods

### Experimental setup

Sliders were manufactured using a Stereolithography (SLA) 3D printer (Formlabs Form 2), with cured resin density *ρ*_*s*_ =  1.15 g/cm^3^. We produced circular sliders with radii in the range 4–12 mm with uncertainty 0.05 mm, elliptical sliders with semi-axes 5.00 ± 0.05 mm and 3.20 ± 0.05 mm, and sliders with radius *R* = 4.00 ± 0.05 mm with grooved bottoms. Masses ranged from *m* = 54 ± 2 mg to 1337 ± 2 mg. The slider mass was increased by adding brass shims and the overall slider thickness never exceeded 1.5 mm. Sliders were coated with a two-part commercial super-hydrophobic spray coating (Ultra-ever Dry)^31^ that allowed the slider to rest stably at the interface and carry large loads without sinking^32–34^. Each slider was embedded with a small permanent magnet of magnetic dipole moment $\overrightarrow{p}$ (Fig. 1(a,d)), which allowed us to guide the slider on a liquid surface by means of an external inhomogeneous magnetic field $\overrightarrow{B}$ through the force ${\overrightarrow{F}}_{B}=(\overrightarrow{p}\cdot \nabla )\overrightarrow{B}$. The magnetic dipole intensity was chosen in order to maximize the slider speed achievable in our experimental setup, while minimizing the influence of ambient magnetic field gradients. We used magnets with magnetic dipole intensity *p* = 0.074 A m^2^ for sliders of radius *R* = 10 mm and 12 mm and magnets with magnetic dipole intensity *p* = 0.019 A m^2^ for all other sliders. Sliders slid on the surface of a water bath in a rectangular glass tank with size 10.2 × 10.2 × 30.5 cm (Fig. 1(a)). The tank was surrounded by a 500-turn coil with inner radius 10.1 cm and outer radius 11.4 cm (Pasco, EM-6723), and its position adjusted such that the coil axis was aligned at the center of the air-water interface. The center of the coil was at a distance 11.5 cm from one of the tank’s short side wall. A small electromagnet was placed behind the same short side walls on the coil axis, and it served to retrieve the slider and fix its initial position. Before each measurement session, the glass tank was cleaned with ethanol and double-rinsed with deionized water. The glass tank was then filled with de-ionized water or de-ionized water-glycerol mixture up to a depth of *h* = 3.7 ± 0.2 cm. The uncertainty on the fluid viscosity was 0.0002 cm^2^/s. The slider was then deposited on the bath’s surface and the tank covered with a transparent lid to avoid ambient contamination. A small opening was left in order to avoid accumulation of vapor on the lid, which would impede the tracking of the slider. The slider deceleration phase was recorded from the top with a CCD camera (Allied Vision Mako U-130B) at 62.5 fps with resolution 1280 × 1024. Electromagnet, coil and camera were controlled by an Arduino and a custom relay-based circuit. In order to avoid magnet re-orientation after switching off the coil, the whole setup was oriented along the Earth’s magnetic field. At least 39 trajectories were recorded per measurement session, in which all the parameters were kept constant. This allowed us to test the repeatability of our setup and measurement and define the variation between runs. The slider center of mass was tracked with a custom object-tracking algorithm implemented in MATLAB. The minimum slider speed considered was *U*_*min*_ = 1 cm/s, one order of magnitude larger than the maximum drift speed we observed as due to the influence of ambient magnetic field gradients, ${U}_{d}\lesssim 0.1$ cm/s.

### Uncertainty on the parameter *α*

Each data set yielded the mean of at least 39 runs, which was fitted with the function $U(t)=\mathrm{1/}{(\mathrm{1/}\sqrt{{U}_{0}}+ct)}^{2}$, where *c* is the fitting parameter. The parameter *α* was then computed as $\alpha =2mc/\rho \sqrt{\nu }\,{R}^{\mathrm{3/2}}$ for circular sliders, and using an analogous formula for elliptical sliders. The uncertainty on *α* was calculated as the square root of the variance formula for independent variables

8 $${\sigma }_{\alpha }=\sqrt{{(\frac{\partial \alpha }{\partial m})}^{2}{\sigma }_{m}^{2}+{(\frac{\partial \alpha }{\partial c})}^{2}{\sigma }_{c}^{2}+{(\frac{\partial \alpha }{\partial \rho })}^{2}{\sigma }_{\rho }^{2}+{(\frac{\partial \alpha }{\partial \nu })}^{2}{\sigma }_{\nu }^{2}+{(\frac{\partial \alpha }{\partial R})}^{2}{\sigma }_{R}^{2}}$$

where *σ*_*c*_ is the value that yields 95% confidence bounds from the fitted value of *c*, *σ*_*m*_ = 2 mg, *σ*_*ρ*_ = 0.002 g/cm^3^, *σ*_*ν*_ = 0.0002 cm^2^/s and *σ*_*R*_ = 0.005 cm.

## Supplementary information

Supplementary information

Supplementary movie
