# Supplementary material for: Friction on water sliders
Source: Sci Rep. 2019 Mar 11;9:4095. doi: 10.1038/s41598-019-40797-y (PMC6411893; doi:10.1038/s41598-019-40797-y)
Supplement: Supplementary file 1 — Supplementary information [file 41598_2019_40797_MOESM1_ESM.pdf]

Supplemental material: Friction on water sliders

Giuseppe Pucci, Ian Ho, and Daniel M. Harris

Brown University, School of Engineering, 184 Hope St., Providence (RI), United States

Supplementary figure

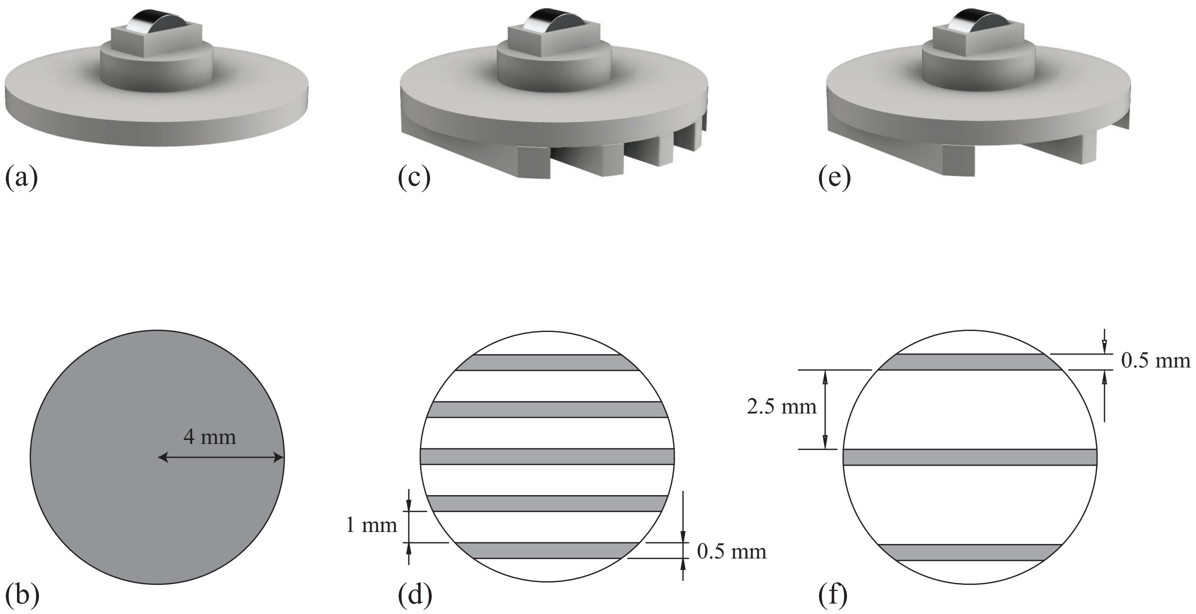

Figure S1: Circular sliders without and with grooved bottoms. (a) 3D drawing of the slider without grooved bottom and (b) plan view of the bottom surface. (c) 3D view of a slider with five ridges and (d) plan view of the bottom surface. (e) 3D view of a slider with three ridges and (f) plan view of the bottom surface. (b, d, f) The area in contact with water is in grey. The sliders radius is 4.00 ± 0.05 mm.

Supplementary table

R (mm)	m (mg)	U_0 (cm/s)	ν (cm^2/s)	α	σ_α
4	61	16.0	0.01	1.72	0.07
6	140	8.7	0.01	1.75	0.04
6	222	8.4	0.01	1.91	0.04
6	305	10.6	0.01	1.77	0.03
8	218	7.3	0.01	1.54	0.03
8	297	11.4	0.01	1.30	0.02
8	388	11.6	0.01	1.44	0.02
8	561	6.1	0.01	2.09	0.03
8	647	7.7	0.01	1.76	0.03
10	396	9.2	0.01	1.62	0.02
10	479	10.4	0.01	1.82	0.03
10	747	9.4	0.01	1.81	0.03
10	830	8.2	0.01	1.88	0.03
10	1091	7.3	0.01	2.07	0.03
12	653	8.6	0.01	1.67	0.02
12	730	9.2	0.01	1.67	0.02
12	992	9.2	0.01	1.48	0.02
12	1074	9.3	0.01	1.53	0.02
12	1337	9.4	0.01	1.68	0.03
10	826	8.2	0.04	1.49	0.02
12	1347	8.0	0.04	1.36	0.01

Table S1: Measured values of α for circular sliders with radius R with uncertainty 0.05 mm, mass m with uncertainty 2 mg and initial speed U_0 with uncertainty 0.1 cm/s, on a fluid bath with kinematic viscosity ν with uncertainty 0.0002 cm^2/s. The uncertainty σ_α was calculated by using the variance formula for independent variables (see "Uncertainty on the parameter α").

Supplementary movie

Sequence of two runs of the experiment. The movie begins with the slider close to the electromagnet, which exerts an attractive force and aligns it along the coil axis. The electromagnet is then turned off while the coil is turned on, thus accelerating the slider along the coil axis through the force $\vec{F}_B = (\vec{p} \cdot \nabla)\vec{B}$, where $\vec{p}$ is the magnetic dipole moment and $\vec{B}$ the magnetic field. Once the slider approximately reaches the center of the coil, the coil is turned off and the slider decelerates due to fluid friction. At the end of the deceleration phase, the coil is momentarily turned on again to redirect the slider toward the electromagnet, which is simultaneously activated to attract the slider to the original position. After all perturbations on the liquid surface disappeared, the slider is accelerated again and the process repeats.
